# Evaluation of the Use of Auricular Composite Graft for Secondary Unilateral Cleft Lip Nasal Alar Deformity Repair

**DOI:** 10.1155/2014/270285

**Published:** 2014-09-25

**Authors:** Percy Rossell-Perry, Carolina Romero-Narvaez

**Affiliations:** ^1^Faculty of Medicine, San Martin de Porres University, Lima, Peru; ^2^Outreach Surgical Center Program Lima Peru, ReSurge International, 120 Schell Street Apartment 1503 Miraflores, Lima 18, Peru; ^3^San Bartolome Children Hospital, Lima, Peru

## Abstract

The purpose of this study is to evaluate the surgical outcome after using composite grafts for secondary cleft lip nasal deformities. A retrospective cohort study of one surgeon's outcome of 35 consecutive performed secondary cleft lip nasal deformity repair. Thirty-five patients with secondary nose deformity related to unsatisfactory cleft lip repair were operated using the proposed surgical technique since 2008. All these patients met the study criterion of having anthropometric measurements performed at least one year postoperatively. Measurement of nostril size was performed at the right and left side of the nose, preoperatively and at least one year postoperatively. The study found statistically significant differences between the preoperatory and postoperatory nose measurements. In addition, we have not found statistically significant differences between the cleft and noncleft nostril sizes measured at least one year postoperatively. The findings suggest that the proposed technique is a good alternative to address secondary nose deformity related to cleft lip primary repair.

## 1. Introduction

Secondary alar deficiencies are a common undesirable outcome after primary unilateral cleft lip nose repair.

Even when this technique has been published previously by many authors, it was not well studied and most of the articles are cases series including a few number of patients.

This problem may be in relation to congenital hypoplasia or surgical technique deficiencies (commonly observed using Millard's subnasal incision).

Proper location of this incision would be difficult to be established in some cases.

**Figure 1:**
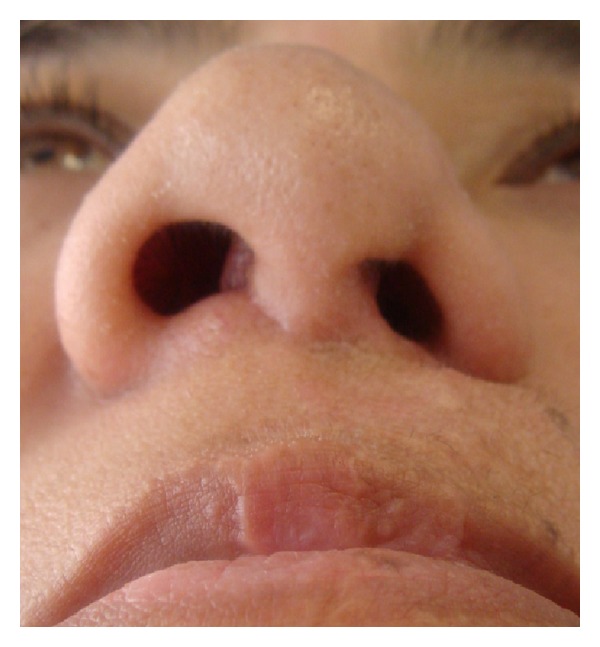
Patient with secondary unilateral cleft lip nose deformity and nose asymmetry related to the use of Millard's subnasal incision.

If the incision is done in a higher position, the alar nose is shortened with the consequent nose asymmetry (Figure 1).

Different techniques have been described for alar reconstruction like local flaps and grafts.

Auricular composite graft is one of the most advantageous methods because it is possible to reconstruct the structural cartilage and skin in one stage.

The use of auricular composite grafts was first described by Koenig in 1902 and the use of two surfaces of skin and cartilage as composite graft was first described by Brown and Cannon in 1946 [1, 2].

Rettinger and O'Connell in 2002 and Ayhan et al. in 2006 described the use of composite grafts for nasal base correction in patients with cleft lip nose in a case series study observing symmetrical and functional results in a small number of patients [3, 4].

Another case series study developed by Cho et al. in 2002 [5] in patients with secondary cleft lip nasal deformity observed an absorption rate of 10% of the graft.

The purpose of this study is to evaluate the symmetry of the nose after using auricular composite grafts for secondary unilateral cleft lip nasal deformities.

## 2. Methods

This is a prospective cohort study of one surgeon's (corresponding author) outcome of 35 consecutive secondary unilateral cleft lip nasal deformity repair from the Outreach Surgical Center Program Lima Peru.

These patients had an open rhinoplasty and the following associated procedures based on Millard Jr. principles [6]:

- previously alveolar bone graft during mixed dentition period,
- vestibular nose lengthening (composite V-Y method) [7],
- medial mobilization of the lateral alar crus (composite V-Y method),
- shortening of the nasal base width (when necessary),
- septoplasty.

All these patients met the study criterion of having anthropometric measurements performed preoperatively and at least one year postoperatively.

**Figure 2:**
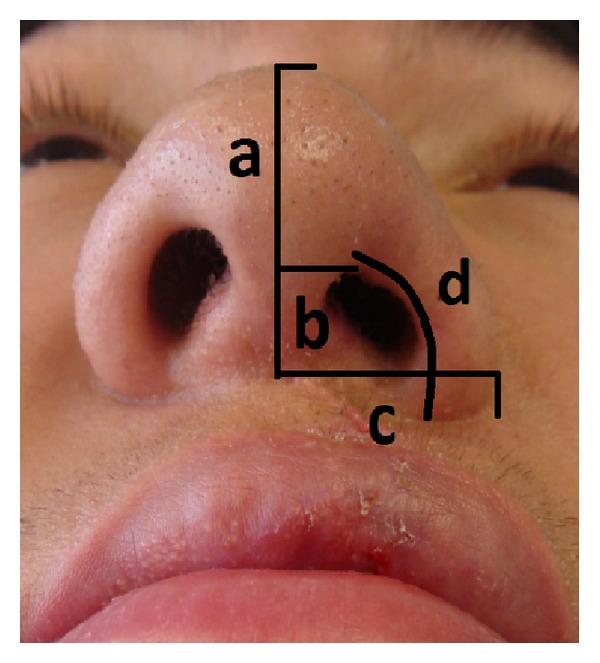
Standard anthropometric measurements. (a) Nostril dome height, (b) nostril apex height, (c) alar width, and (d) alar length.

These were the standard anthropometric measurements (Figure 2):

- nostril dome height, which was measured from the midway point at the base of the columella to the highest point on the nasal dome;
- nostril apex height, which was measured from the midway point at the base of the columella to the highest point of the nostril;
- alar width, which was measured from the midway point at the base of the columella to the most lateral point of the nostril in a line perpendicular to the axis of the columella;
- alar length, measured from the alar columellar junction to the alar base.

Measurements were performed at the right and left side of the nose, preoperatively and at least one year postoperatively.

These measurements were compared in order to determine pre- and postoperatively nose symmetry.

Outcomes were additionally determined by a parent questionnaire assessment.

Differences in alar length were identified in these patients in order to evaluate the amount of tissue necessary for alar reconstruction.

The tissue's requirement is estimated using the length of the alar nose measured from the alar-columellar junction to the alar base.

Based on our experience, the graft is designed 50% larger than the estimated defect due to the scar contracture of the graft observed during the healing process.

In order to maximize graft survival, careful preoperative design, preparation of the recipient site, meticulous surgical technique, and diligent postoperatory care are mandatory.

The recipient bed of the graft located at the deficient alar base must be in good condition for proper development of the process of plasmatic imbibition, vascular inosculation, and neovascularization.

All the scar tissue should be excised from the recipient site leaving a healthy raw surface to receive the auricular composite graft. The use of electrocautery should be avoided as possible.

### 2.1. Surgical Technique

**Figure 3:**
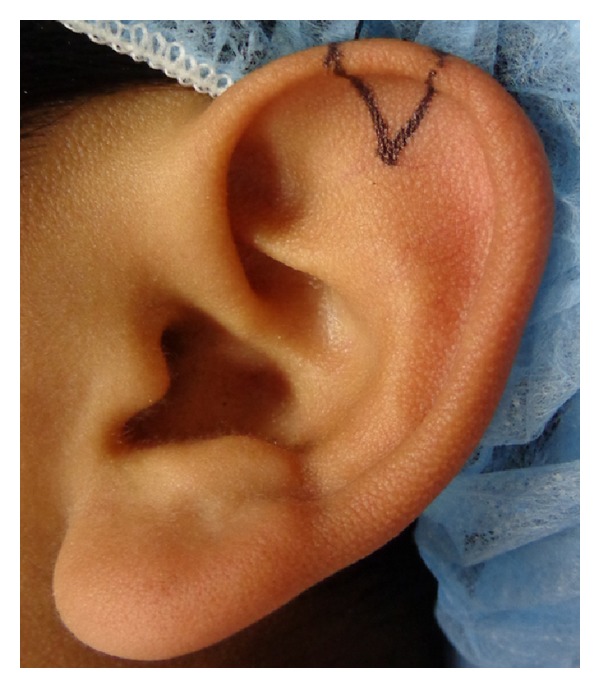
Designed area of the required auricular tissue from the helix.

After design with marking pen of the required auricular tissue in the ear, local anesthetic without epinephrine is injected around the designed composite graft avoiding any hydrodissection of the skin off the cartilage (Figure 3).

The skin and cartilage are incised in one block with 15C blade following the skin markings.

**Figure 4:**
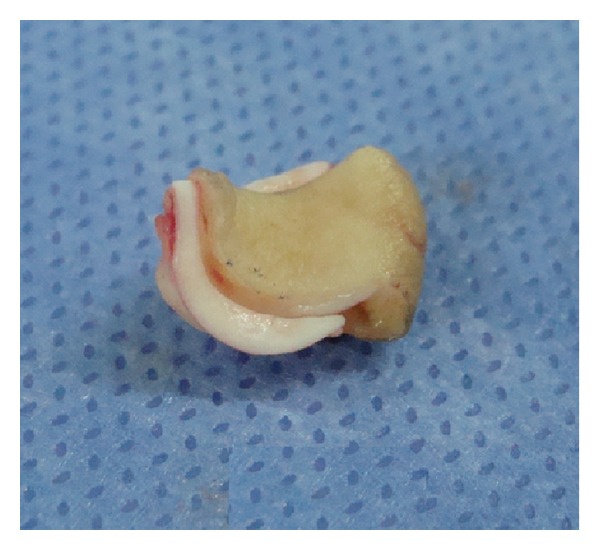
Auricular composite graft. The size of the cartilage component should be a few millimeters bigger than the skin component. This is a 15 × 18 mm composite graft.

The size of the cartilage component should be a few millimeters bigger than the skin component in order to guarantee their integrity as composite graft (Figure 4).

The graft should be harvested carefully and manipulated gently grabbing the cartilage and skin with the forceps simultaneously. Any skin traction alone may separate it from the cartilage losing the tissue connection between them.

After this, carefully edge to edge closure is performed at the donor site using skin stitches.

Up to 1.5 centimeter defect can be closed primarily without problems. Larger grafts are rarely required for secondary alar nose repair in these patients.

**Figure 5:**
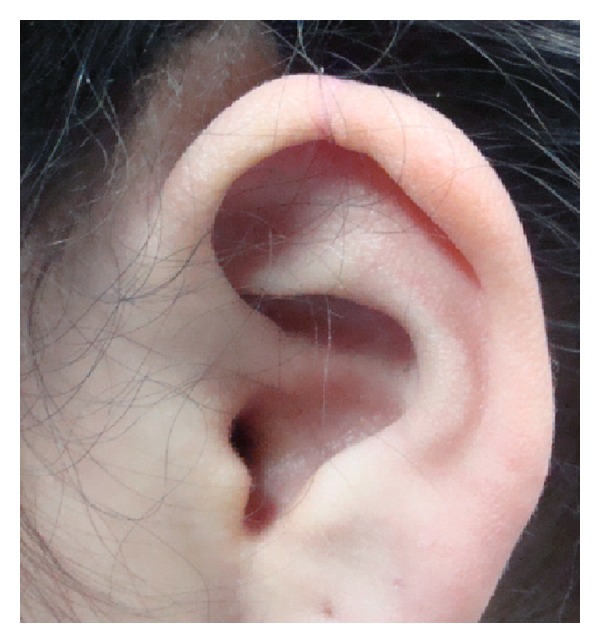
Long term appearance of the donor site after 18 months.

Long term appearance of the donor site is acceptable (Figure 5).

The affected alar nose is incised at the level of the scar and this scar tissue should be removed carefully leaving healthy tissue at the borders to receive the composite graft and guarantee its survival. Use of cautery should be avoided as possible.

The skin edges of the auricular composite graft are directly closed to the defect's skin edges with simple resorbable sutures.

The composite graft is located between the alar nose and upper lip suturing the skin component only using resorbable simple stitches.

Stitches are placed first at the corners (internal and external) and between the graft and alar nose and upper lip, making easier the application of the following sutures. These stitches should not include the cartilage component and must be placed superficially in order to avoid any bad alignment between the borders.

Special attention must be taken with the sutures located around the medial surface of the graft where the skin is firmly attached to the cartilage. At this level the stitches should include only the skin avoiding any disruption between the component's graft.

Antibiotic ointment is applied to the surgical surfaces.

Moisturizing ointment is recommended to be applied over the graft during one week in order to avoid graft desiccation (greatest risk for graft failure).

Appearance of the grafts is not good during the first days becoming first blue and then pink after epidermolysis. Complete survival was confirmed after 7 days.

We did not use any drug to improve graft survival in the studied group.

### 2.2. Statistical Analysis

A two-sample test of proportions was performed to assess the statistical significance between the two methods. *P* < 0.05 yielded a confidence level of 95%.

The data were analyzed using Stata 11.0 software.

## 3. Results

Thirty-five patients with secondary alar nose deformity related to unsatisfactory unilateral cleft lip repair were operated using the proposed surgical technique since 2008.

The mean age at the time of the surgery was 11.57 years (range 8 to 14 years).

 Gender: men 24 (68.57%) and women 14 (31.43%).
 Side: left 23 (65.71%) and right 12 (34.29%)

The preoperatively mean height and width of auricular composite tissue were 16.21 mm (range: 18 to 13 mm) and 10.35 mm (range: 13 to 8 mm), respectively.

We observed statistically significant preoperatory differences between the cleft and noncleft sides and between the preoperatory and postoperatory nose measurements (Tables 1 to 3).

In addition, we have not found statistically significant differences between the cleft and noncleft sides measured at least one year postoperatively (Table 2).

Observed rate of graft survival was 100% with 2 partial necroses.

90.35% of the patients were very satisfied with the surgical outcomes.

Three patients (8.5%) showed fair results with some recurrence of the asymmetry of both nostrils.

Surgical outcomes are presented in Tables 1 to 3 and Figures 6, 7, 8, 9, 10, 11, 12, and 13.

## 4. Discussion

Surgical repair of the nasal deformity caused by trauma, tumor extirpation, burns, and cleft lip nose is challenging because it requires reconstruction of the outer and inner skin and supporting cartilage [3–5, 8–12].

The auricular composite graft let us reconstruct the three-layered structure of the alar and has a similar shape, curve, color, and texture. In addition, the graft is similar to the recipient site because the ear has components with various shapes and curves.

Primary closure of the donor site can be achieved for a defect less than 15 mm as described by Singh and Bartlett in 2007 [13] (Figures 4 and 5).

In case of secondary cleft lip nasal deformity with severe tissue deficiency, auricular composite graft can be useful for columellar lengthening or for creating symmetrical nostrils [10–12].

The main disadvantage of this method is the contracture of the graft after the healing process which has been observed in all cases.

Difference between pre- and postoperative width of the graft was 5.27 mm and represents 50.91% of the initial width of the graft (10.35 mm).

This percentage represents the contracture of the graft and supports our preoperatory design considering a composite graft 50% larger than the estimated defect.

This outcome is similar to the initial report made by Rees [14] with 53% of reabsorption.

Grafted tissues frequently shrink their width and thickness. In order to improve this situation, we design the composite grafts larger than the estimated defect preoperatively.

Cosmetic appearance of the graft is difficult to be improved and this is the main limitation of this technique.

In relation with the size of the graft, initial studies concluded that a graft larger than 10 mm results in subsequent necrosis of the graft [2, 15].

However, we use larger grafts (mean 20 × 12 mm) with high rate of survival (85.71%).

Parkhouse and Evans [16] reported similar results with a successful graft of 10 × 18 mm for alar reconstruction.

Previous anthropometric studies described by Farkas et al. [17] did not conclude major differences in relation to the age (range: 6 to 13 years old) and gender.

This is the reason why these variables are not affecting our results and any stratification was not necessary in the studied group.

Many papers have been published about the use of auricular composite graft in aesthetic and reconstructive surgery.

Most of them are case series studies including a small number of patients.

This is the first analytic research to evaluate objectively differences between the cleft and noncleft side of the nose pre- and postoperatively.

Objective evaluation of the cleft nose deformity after using conventional Millard's technique for primary repair has been done and nostril apex height, alar length, and width asymmetry have been determined in all the cases preoperatively (Table 1).

**Figure 6:**
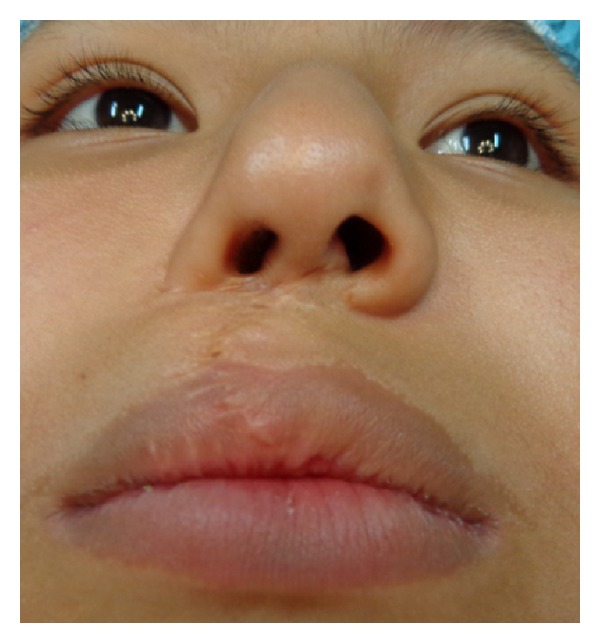
Case 1: A 9-year-old female patient with a right unilateral cleft lip initially treated using conventional Millard technique. At this time the nose is asymmetric with deficiency of the right alar nose.

**Figure 7:**
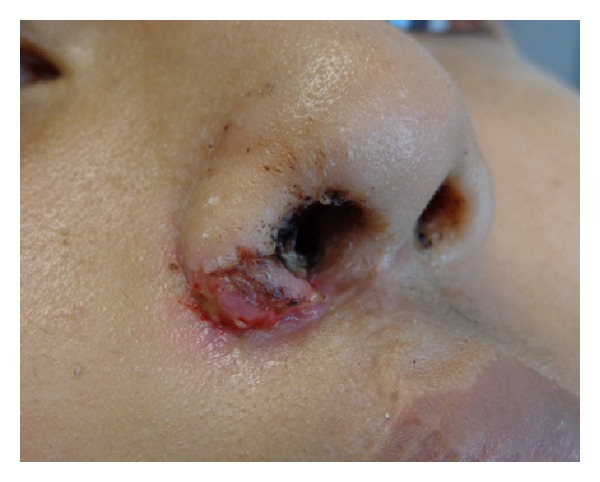
The patient is shown one week after secondary rhinoplasty using auricular composite graft. The graft is having some epidermolysis.

**Figure 8:**
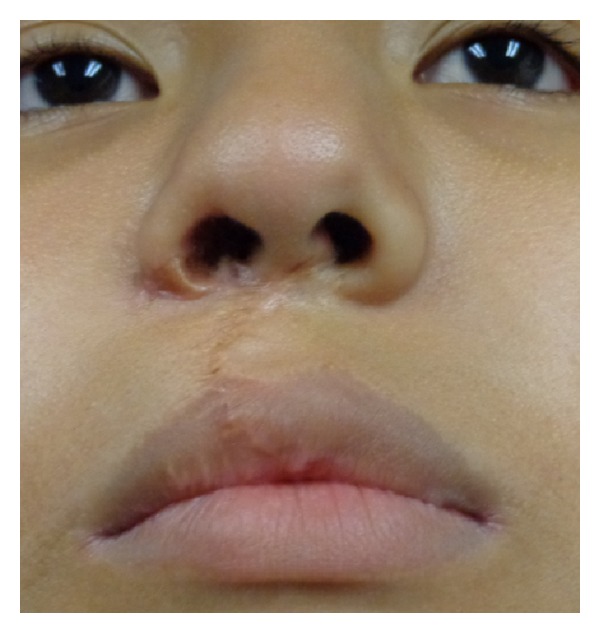
Postoperative view of the patient two years after surgery.

**Figure 9:**
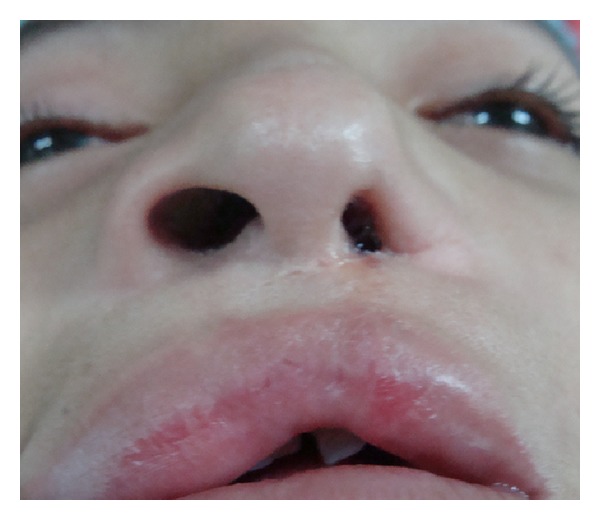
Case 2: A 12-year-old female patient with a left unilateral cleft lip initially treated using conventional Millard technique. At this time the nose has a severe deformity with functional impairment and nose asymmetry.

**Figure 10:**
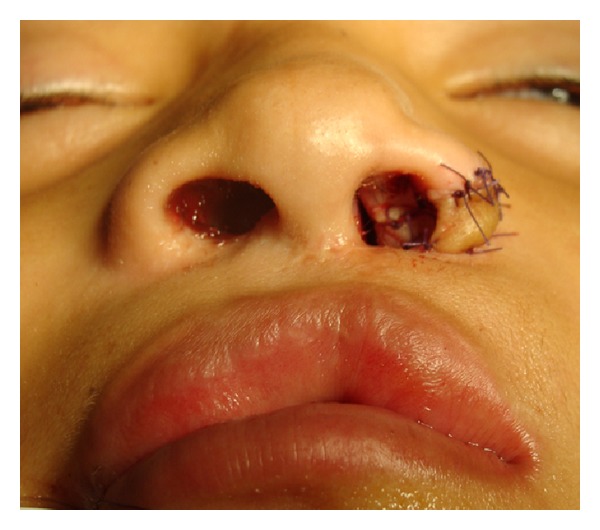
Intraoperative image shows nose asymmetry using the auricular composite graft for nose repair.

**Figure 11:**
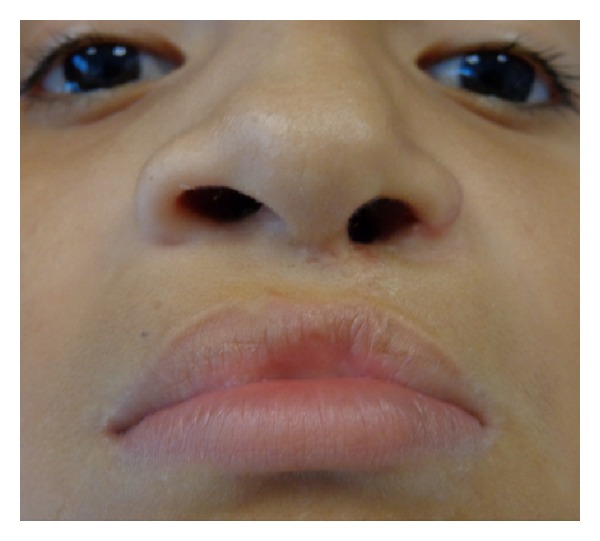
Postoperative view of the patient three years after surgery illustrating cosmetic and functional improvement of the nose with small contracture of the graft after the healing process.

**Figure 12:**
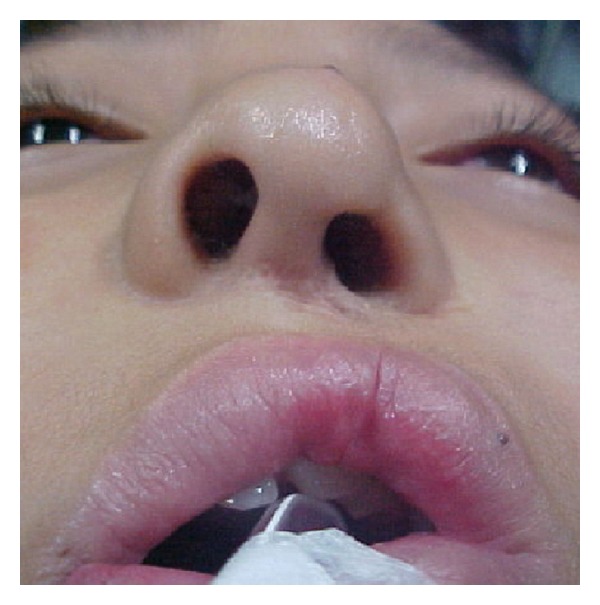
Case 3: A 10-year-old female patient with a left unilateral cleft lip initially treated using conventional Millard technique. The nose has a severe deformity with nose asymmetry.

**Figure 13:**
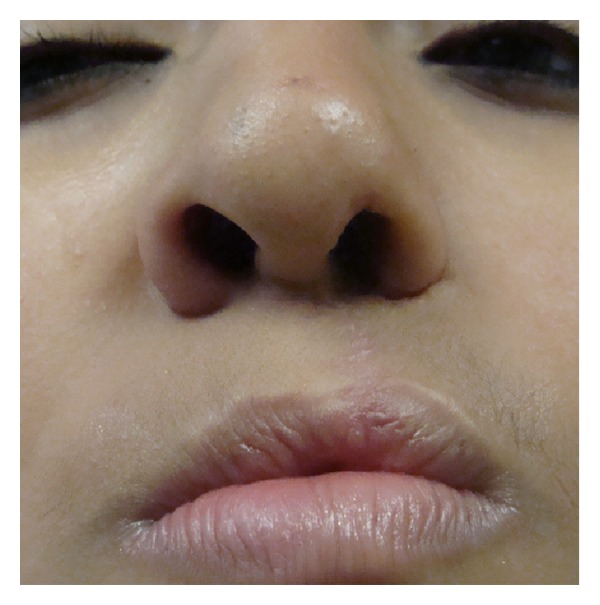
Postoperative view of the patient four years after surgery.

Effectiveness of the proposed technique for secondary nose deformity repair has been confirmed with this comparative study observing nonstatistical significant differences between the cleft and noncleft side and statistical significant differences between pre- and postoperatively anthropometric nose measurements (Tables 2 and 3) (Figures 6 to 13).

We observe some small differences between the cleft and noncleft side in the presented cases; however, they are small and nonstatistically significant. These differences are mainly related to the recurrence of the septal deviation.

An adequate and symmetrical nasal tip projection was obtained by repositioning the cleft lower lateral cartilage; however, these were not statistically significant (*P*: 0.14) (Table 3).

Nasal retainers are commonly used in cleft nose deformity repair; however, we did not use any molding device in this study due to the observed complications related to their use, like skin reaction, infection, ulceration, recurrence, pain, and others.

This problem requires additional studies to evaluate cosmetic appearance of the graft.

## 5. Conclusions

The proposed technique of open rhinoplasty in combination with auricular composite grafts has been useful for gaining additional alar length in patients who otherwise have a satisfactory relationship between the anatomic subunits.

The findings suggest that the proposed technique is a good alternative to address secondary nose deformity related to unilateral cleft lip primary repair.

## Figures and Tables

**Table 1:** Comparisons of cleft side and noncleft side using auricular composite graft technique preoperatively by the Outreach Surgical Center Program Lima 2008 to 2013.

Nose segment	Noncleft	Cleft	*P*	CL
	*n*: 35	*n*: 35		
	Mean	Mean		
Nostril apex height	13 ± 1.22	8.82 ± 1.78	0.0003	−4.18 (−6.08; −2.29)
Alar width	17.33 ± 2.66	21.92 ± 1.61	0.0002	4.59 (2.53; 6.65)
Alar length	20 ± 2.57	14.94 ± 1.06	0.00001	−5.06 (−5.45; −2.66)

**Table 2:** Comparisons of cleft side and noncleft side using auricular composite graft technique at 1 year or more postoperatively by the Outreach Surgical Center Program Lima 2008 to 2013.

Nose segment	Noncleft	Cleft	*P*	CL
	*n*: 35	*n*: 35		
	Mean	Mean		
Nostril apex height	13 ± 1.22	11 ± 0.58	0.05	2 (0.02; 3.99)
Alar width	17.33 ± 2.66	18.11 ± 2.86	0.21	0.78 (−0.54; 2.09)
Alar length	20 ± 2.57	20.21 ± 1.76	0.41	−0.21 (−2.74; 1.17)

**Table 3:** Comparisons of cleft side pre- and postoperative nose measurements after 1 year using the proposed technique in patients with secondary unilateral cleft lip nose deformity by the Outreach Surgical Center Program Lima 2008 to 2013.

Nose segment	Preoperatory	Postoperatory	*P*
	*n*: 35	*n*: 35	
	Mean	Mean	
Nostril apex height	8.82 ± 1.78	11 ± 0.58	0.004
Alar width	21.92 ± 1.61	18.11 ± 2.86	0.001
Alar length	14.94 ± 1.06	20.21 ± 1.76	0.00001
Nostril dome height	19.31 ± 1.65	20.67 ± 2.07	0.14

## References

[1] Koenig F (1902). Zur deckung von defekten der nasenflügel. *Berl Klein Wochenschr*.

[2] Brown JB, Cannon B (1946). Composite free grafts of two surfaces of skin and cartilage from the ear. *Annals of Surgery*.

[3] Rettinger G, O'Connell M (2002). The nasal base in cleft lip rhinoplasty. *Facial Plastic Surgery*.

[4] Ayhan M, Gorgu M, Erdogan B (2006). Various applications of chondrocutaneous composite grafts in secondary cleft lip nose patients. *Journal of Craniofacial Surgery*.

[5] Cho BC, Park JW, Baik BS (2002). Correction of severe secondary cleft lip nasal deformity using a composite graft: current approach and review. *Annals of Plastic Surgery*.

[6] Millard DR (1982). Earlier correction of the unilateral cleft lip nose. *Plastic and Reconstructive Surgery*.

[7] Potter J (1954). Some nasal tip deformities due to alar cartilage abnormalities. *Plastic and Reconstructive Surgery*.

[8] Gurunluoglu R, Shafighi M, Gardetto A, Piza-Katzer H (2003). Composite skin grafts for basal cell carcinoma defects of the nose. *Aesthetic Plastic Surgery*.

[9] Son D, Kwak M, Yun S, Yeo H, Kim J, Han K (2012). Large auricular chondrocutaneous composite graft for nasal alar and columellar reconstruction. *Archives of Plastic Surgery*.

[10] Cheon YW, Park BY (2010). Long-term evaluation of elongating columella using conchal composite graft in bilateral secondary cleft lip and nose deformity. *Plastic and Reconstructive Surgery*.

[11] Saha S, Kumar V, Kashanchi R, Agrawal A (2005). Correction of the nose in patients with unilateral cleft lip with composite grafts. *Scandinavian Journal of Plastic and Reconstructive Surgery and Hand Surgery*.

[12] Matsuo K, Hirose T (1990). Secondary correction of the unilateral cleft lip nose using a conchal composite graft. *Plastic and Reconstructive Surgery*.

[13] Singh DJ, Bartlett SP (2007). Aesthetic management of the ear as a donor site. *Plastic and Reconstructive Surgery*.

[14] Rees TD (1960). The transfer of free composite grafts of skin and fat: a clinical study. *Plastic and Reconstructive Surgery*.

[15] Lehman JA, Garrett WS, Musgrave RH (1971). Earlobe composite grafts for the correction of nasal defects.. *Plastic and Reconstructive Surgery*.

[16] Parkhouse N, Evans D (1985). Reconstruction of the ala of the nose using a composite free flap from the pinna. *British Journal of Plastic Surgery*.

[17] Farkas LG, Forrest CR, Phillips JH (2000). Comparison of the morphology of the “cleft face” and the normal face: defining the anthropometric differences. *Journal of Craniofacial Surgery*.

